# Hydrophobic interface layer improves the moisture tolerance and efficiency of ambient air-processed perovskite solar cells

**DOI:** 10.1016/j.fmre.2024.12.013

**Published:** 2024-12-27

**Authors:** Wanjie Yin, Huiming Luo, Ligang Yuan, Yuxuan Sun, Xiao Yang, Longyan Zhang, Yong Peng, Qing-Song Jiang

**Affiliations:** a Faculty of Electronic Information Engineering, Huaiyin Institute of Technology, Huai'an 223003, China; b Institute for Materials Discovery, University College London, Malet Place, London, WC1E 7JE, UK; c Key Laboratory for Optoelectronic Information Perception and Instrumentation of Jiangxi Province, Key Laboratory of Nondestructive Testing Ministry of Education, School of the Testing and Photoelectric Engineering, Nanchang Hangkong University, Nanchang 330063, China; d State Key Lab of Advanced Technology for Materials Synthesis and Processing, Wuhan University of Technology, Wuhan 430070, China

**Keywords:** Perovskite solar cells, Ambient air fabrication, Poly(N-vinylcarbazole), Surface passivation, Hydrophobic property

## Abstract

Because perovskite films degrade quickly in humidity conditions, perovskite solar cells (PSCs) prepared in ambient air still show lower photovoltaic performance than those prepared in an inert atmosphere. Here, we offer an effective interfacial passivation method via a poly(N-vinylcarbazole) (PVK) layer to protect perovskite films and prevent their moisture degradation under ambient air conditions. The power conversion efficiency of the PVK-based champion PSC is 23.27% and is higher than that of the control PSC (20.94%). Meanwhile, the PVK-based perovskite films exhibit a large water contact angle, which is attributed to the hydrophobic nature of the uniformly deposited PVK layer. This leads to no significant effect for the PVK-based perovskite film with moisture degradation for 6 days under an RH of 50%, and the corresponding PVK-based PSC exhibits a higher efficiency of 21.64% and a smaller hysteresis index. Moreover, even after 3550 h of storage in an argon glovebox, the PVK-based PSC retains 98% of its initial efficiency.

## Graphical abstract

FA_0.95_Cs_0.05_PbI_3_ perovskite films are fabricated in ambient air with RH of 40–60% and passivated by a poly(N-vinylcarbazole) (PVK) layer. The corresponding perovskite solar cell exhibits the small non-radiative open-circuit voltage loss (102 mV). The champion efficiency of perovskite solar cells achieves 23.27%, which sustains 98% of the initial PCE after about 3550 h in an argon-filled glovebox.

**Figure:**
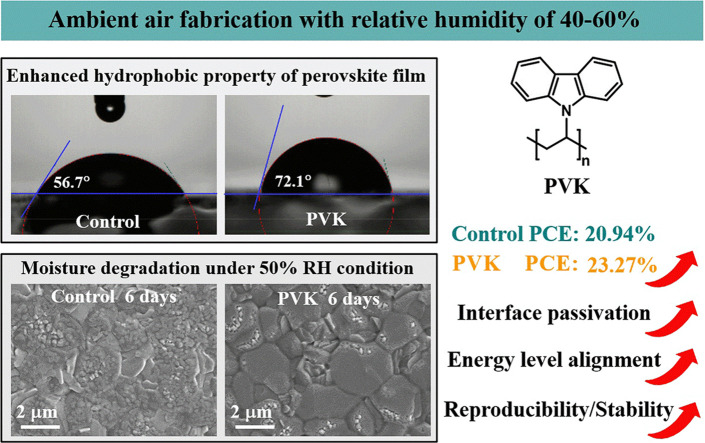

## 1 Introduction

Perovskite materials, known for their broad absorption spectrum, tunable band gap, and high carrier mobility, have been widely explored and applied in photodiodes, photocatalysts, solar cells, and photodetectors [[1], [2], [3], [4]]. In particular, perovskite solar cells (PSCs) have attracted immense attention since they were first reported in 2009 [5]. So far, PSCs have obtained a record power conversion efficiency (PCE) of over 26% [6]. Although PSCs with high photovoltaic performance exhibit great potential for future commercialization, the fabrication and storage of ideal perovskite films highly rely on an inert environment [7,8]. Considering the requirements of potential large-scale industrialization, it is very urgent and important to explore efficient and stable PSCs in the air environment [9].

The presence of water molecules is the main challenge in the fabrication process of PSCs under ambient air environments. It can disrupt the formation process of perovskite film and lead to the decomposition of the perovskite crystals at their surface or grain boundaries [10,11]. Effective control of the crystallization quality of the perovskite film is crucial to counteract the corrosive effect of water molecules [[12], [13], [14], [15]]. For example, both PbS quantum dots and methylammonium chloride (MACl) were incorporated into a perovskite precursor solution to fabricate a FAPbI_3_ film under ambient air conditions of 40–60% relative humidity (RH), resulting in a champion PCE of 19.4% for the corresponding PSCs [12]. Similarly, Pb(SCN)_2_, PEASCN, and MASCN were used as additives to modulate MAPbI_3_ films with homogeneous morphology and high crystallization in the ambient air fabrication process [[16], [17], [18]]. The quality of CsPbI_3_ perovskite films was also improved by using multifunctional ethacridine lactate as an additive [19]. The corresponding PSCs have improved long-term stability in ambient air without encapsulation.

Furthermore, significant efforts have been made to improve the surface quality of perovskite films prepared in ambient air. Surface modulation of the perovskite film is a universal strategy to improve the PCE of PSCs by passivating the surface defects and aligning the energy band between the perovskite and the charge transport layer [20,21]. 3-fluoro-benzyl ammonium iodide was used as the bulky organic cation to form a high-quality quasi-2D perovskite film, resulting in a remarkable PCE of 20.12% for the corresponding PSCs [22]. Two-dimensional Dion-Jacobson phase perovskite with stable structure was obtained by introducing 4-(aminomethyl)piperidine iodide on the surface of the three-dimensional perovskite in ambient air, which also facilitated charge transfer and improved the open-circuit voltage. The corresponding PSC achieved a high PCE of 20.11% and had 95% of the initial PCE after 700 h of storage in ambient air with 30–40% RH [23]. The three-dimensional perovskite films were also post-treated with cyclohexylmethylammonium iodide to form a hydrophobic surface and enhance the reproducibility of high-efficiency PSCs under ambient air fabrication conditions with a wide humidity window [24]. Therefore, the decomposition of perovskite grains at high RH is effectively suppressed by the surface modulation strategy.

An effective surface passivation material is n/p type polymers with multiple functional groups [25]. The interface between TiO_2_ and MAPbI_3_ films was modified with an ultra-thin polyaniline layer, which is beneficial for suppressing the charge state of a Pb^2+^ defect in ambient air [26]. Meanwhile, the surface of MAPbI_3_ films was also passivated by 4‑tert-butylpyridine to improve the moisture stability of PSCs, due to their hydrophobic properties [27]. The perovskite films could be protected by introducing the light-induced cross-linking of acrylamide monomers, resulting in reduced trap density through carboxyl-group coordination [28]. Then, the polymer with hydrophobic properties displays an important role in the fabrication process of PSCs under ambient air conditions. It is well known that poly(N-vinylcarbazole) (PVK) with hydrophobic property has been developed to passivate defects of MAPbI_3_ films in N_2_-filled glove box and facilitate carrier transfer at MAPbI_3_/spiroOMeTAD interfaces by the carbazole functional group [29,30]. Furthermore, PVK shows a deep HOMO energy level, which is beneficial to lessen carrier accumulation and further encourage carrier transfer [29]. Therefore, it is urgent to explore the interaction mechanism between PVK and perovskite films prepared in ambient air.

In this work, PVK is chosen as a surface hydrophobic material to shield the surface of perovskite films under ambient air conditions. PVK can passivate the defects on the perovskite films and facilitate the charge transfer at the interface between the perovskite film and the spiro-OMeTAD layer. As a result, the PCE of the PVK-based PSCs prepared in ambient air achieves 23.27% at a PVK concentration of 2 mg mL^−1^. At the same time, the water contact angle on the surface of the PVK-based perovskite films increases from 56.7° to 72.1°. There are no significant changes from SEM measurements for the PVK-based perovskite films degraded in an environment with an RH of about 50% for 6 days. The corresponding PVK-based PSC still exhibits a high PCE of 21.64%, demonstrating a good humidity tolerance because of its hydrophobic nature.

## 2 Material and methods

### 2.1 Materials

Formamidinium iodide (FAI, 99.5%) was purchased from Greatcell Solar Ltd. Lead (II) iodide (PbI_2_, 99.99%), methylammonium chloride (MACl, 99.5%), 4‑tert-butylpyridine (tBP, > 96%), *2, 2′, 7, 7′*-tetrakis[N*,*N-di(4-methoxyphenyl)amino]- *9, 9*′-spirobifluorene (Spiro-OMeTAD, ≥ 99.5%) and lithium bis(trifluoromethanesulphonyl) imide (LiTFSI, 99%) were purchased from Xi'an Yuri Solar Co., Ltd. Cesium iodide (CsI, 99.999%), PVK (molecular weight is 1,100,000), N*,* N-dimethylformamide (DMF, 99.8%), dimethyl sulfoxide (DMSO, 99.8%), chlorobenzene (CB, 99.8%), isopropyl alcohol (IPA, 99.5%), and acetonitrile (ACN, 99.9%) were purchased from Sigma-Aldrich. SnO_2_ colloid solution (15% in H_2_O colloidal dispersion) was purchased from Alfa Aesar. The above reagents were used as received.

### 2.2 Device fabrication

The washed indium-doped tin oxide (ITO) glasses were treated with ultraviolet ozone for 30 min after annealing at 150 ℃ for 12 h. Firstly, a SnO_2_ film was formed by spin-coating SnO_2_ solution with a weight fraction of 2.67% onto ITO glass and annealing at 150 °C for 30 min, which was prepared by diluting the SnO_2_ colloid solution with ultra-pure water. Secondly, the FA_0.95_Cs_0.05_PbI_3_ perovskite film was fabricated in ambient air with 40–60% RH by a two-step deposition process. The SnO_2_ film was treated with ultraviolet ozone for 30 min and transferred to a spin-coater. A PbI_2_ solution was prepared by dissolving 360.0 mg of PbI_2_ and 10.2 mg of CsI in 600 µL of the mixed solvent (volume ratio DMSO: DMF of 5:95) and spin-coated on a SnO_2_ film at 2000 rpm for 50 s. At the end of the 20 s spin-coating time, 40 µL of FAI solution was rapidly dropped onto the spinning film, which was prepared by dissolving 78.0 mg of FAI and 15.2 mg of MACl in 1.3 mL of isopropanol. The wet film was then transferred to a hot plate and annealed at 150 °C for 10 min. For interfacial passivation, PVK solution (1, 2 and 4 mg mL^−1^ in CB) was spin-coated onto the perovskite film at 3000 rpm for 30 s. Thirdly, the hole transport layer was fabricated by spin-coating spiro-OMeTAD precursor solution onto the perovskite film for 30 s at 3500 rpm under ambient air conditions with 20% RH, which was obtained by dissolving 72.3 mg of spiro-OMeTAD, 28.8 µL of tBP, and 17.5 µL of LiTFSI solution (520 mg of LiTFSI in 1 mL of ACN) in 1 mL of CB. Finally, an 80 nm Ag film was thermally vapor deposited as a metal electrode using a shadow mask. The active area of the PSCs was 8 mm^2^.

### 2.3 Characterization

The scanning electron microscope (SEM) images of perovskite films and PSCs were characterized by Zeiss Merlin. The absorption spectra of perovskite films were obtained by the UV–Vis-NIR spectrophotometer (UV3200). The static contact angles of FA_0.95_Cs_0.05_PbI_3_ perovskite films were detected by a contact angle measurement system (OCA15, Dataphysics, Germany). The X-ray diffraction (XRD, D8 DISCOVER) was used to analyze the phases of perovskite films. Fourier transform infrared spectra (FTIR, CCR-1, Thermo Nicolet) were used to analyze the interaction between PVK and PbI_2_ through characteristic peaks of functional groups. The ultraviolet photoelectron spectroscopy (UPS, Thermo Nexsa G2) cooperated with the emission energy (21.22 eV) of Helium irradiation was used to measure the energy level of perovskite films. The photoluminescence (PL) spectra of perovskite films were measured by the PL system. A continuous-wave solid-state laser with a wavelength of 405 nm was used as the excitation pump. Time-resolved photoluminescence (TRPL) spectra of perovskite films were collected by FLS1000 (Edinburgh Instrument, UK) with an excitation wavelength of 450 nm. The external quantum efficiency (EQE) of PSCs was tested by the Zahner system in combination with a TLS03 light source (300 Hz, 100 counts) on an IM6 electrochemical workstation (Zahner Zennium, Germany). Electrochemical impedance spectra (EIS) and Mott-Schottky analysis of PSCs were also characterized by the IM6 electrochemical workstation. A standard solar simulator (SS-F5–3A, Enli Technology CO., Ltd.) was used to provide an AM 1.5 G solar spectrum. The current density-voltage (J-V) curves of PSCs were obtained by a Keithley 2400 digital source meter at 25 ℃. The luminance and electroluminescence (EL) spectra of PSCs were recorded by a luminance meter (Konica Minolta, CS-200) and a Flame spectrometer (Ocean Optic), respectively. The hole-only devices (ITO/PEDOT:PSS/perovskite/spiro-OMeTAD/MoO_3_/Ag) were prepared and measured by the dark J-V curves, which was used to analyze the effect of PVK by the space-charge-limited current (SCLC) method. The unencapsulated PSCs were stored in an argon-filled glovebox and measured at regular intervals for long-term stability. The photothermal stability of the unencapsulated PSCs was evaluated by using a white light-emitting diode (LED) lamp at the maximum power point (MPP).

## 3 Results and discussion

**Fig. 1:**
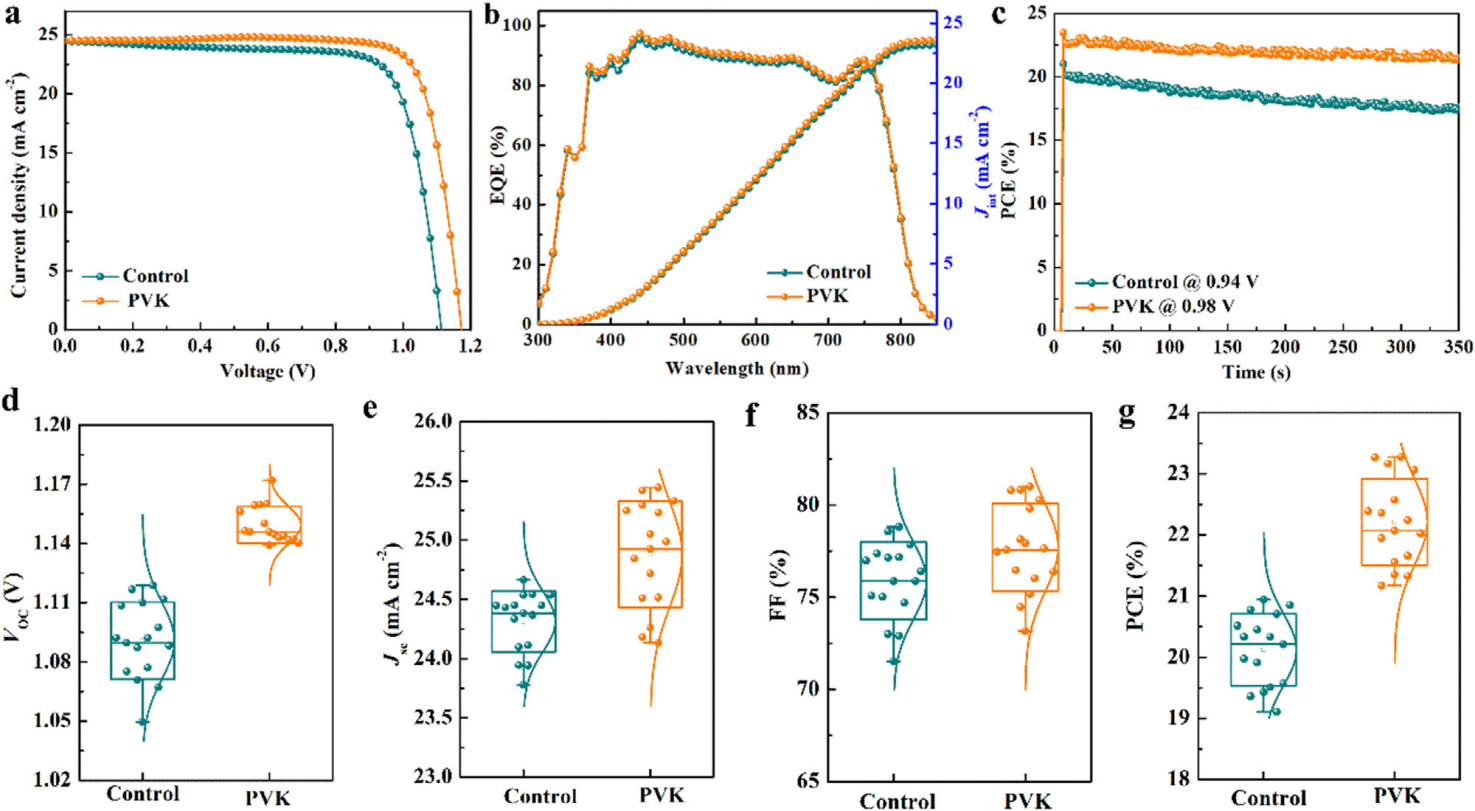
(a) J-V curves, (b) EQE spectra and integrated *J*_sc_, and (c) steady-state output power of PSCs without and with PVK layer. The statistical distribution of photovoltaic parameters of PSCs without and with PVK layer: (d) *V*_oc_, (e) *J*_sc_, (f) FF, and (g) PCE.

The PSCs with a typical structure of ITO/SnO_2_/FA_0.95_Cs_0.05_PbI_3_/PVK/spiro-OMeTAD/Ag are selected and fabricated in ambient air with a RH of 40–60% and a temperature of 25–35 ℃. The FA_0.95_Cs_0.05_PbI_3_ perovskite films are fabricated using a two-step deposition process [31]. PVK solutions with different concentrations are spin-coated onto FA_0.95_Cs_0.05_PbI_3_ perovskite films to passivate surface defects and avoid water degradation of perovskite films under ambient air conditions during the fabrication process. The effect of the PVK solution concentration on the photovoltaic performance of PSCs is investigated by J-V curves, as shown in Fig. S1. The photovoltaic parameters of the corresponding PSCs are summarized in Table 1. It is noteworthy that the optimized PVK solution concentration is 2 mg mL^−1^. From Fig. 1a and Table 1, the champion PSC based on the PVK layer exhibits the highest PCE of 23.27%, higher than that of the control device (20.94%). Compared with the relative reports, the PVK-based PSC exhibits a high photovoltaic performance [[26], [27], [28],^32^]. In addition, the hysteresis index (HI) of PSCs is studied and shown in Fig. S2 and Table S1. The HI of the champion PVK-based PSC decreased to 4.6% from 11.5% of the control device. The suppression of the hysteresis phenomenon can be attributed to the more efficient hole extraction and transport, resulting from improved interfacial contact and the passivation of the defects at the surface and grain boundaries [33,34]. To verify the reliability of device efficiency, *J*_sc_ values of PSCs are calculated by integrating the EQE spectra, as shown in Fig. 1b. The calculated *J*_sc_ values of PSCs without and with PVK layer are 23.18 mA cm^−2^ and 23.55 mA cm^−2^, respectively. This agreement between the EQE spectra and the J*-*V curves confirms the consistency of the experimental results [35]. Fig. 1c shows the steady-state performance of PSCs without and with the PVK layer. The PVK-based PSC exhibits steady-state output power for 350 s measured at an applied voltage of 0.98 V, proving the improved stability of the PVK-based PSC.

**Table 1:** **Photovoltaic parameters of PSCs based on PVK layers with different concentrations in reverse scan measurement**.

PSCs	*V*_oc_ (V)	*J*_sc_ (mA cm^−2^)	FF (%)	PCE (%)
Control	1.11	24.45	77.16	20.94
PVK with 1 mg mL^−1^	1.13	24.21	78.19	21.39
PVK with 2 mg mL^−1^	1.17	24.51	81.15	23.27
PVK with 4 mg mL^−1^	1.16	24.71	76.79	22.01

Moreover, the statistics of photovoltaic performance for 16 PSCs without and with PVK are shown in Fig. 1d-g and summarized in Table S2. The average PCE of PSCs increases from 20.12 ± 0.59% to 22.21 ± 0.71% (from 24.31 ± 0.26 mA cm^−2^ to 24.88 ± 0.45 mA cm^−2^ for the average short-circuit current density (*J_SC_*), from 1.09 ± 0.02 V to 1.15 ± 0.01 V for open-circuit voltage (*V_OC_*), and from 75.89 ± 2.09% to 77.69 ± 2.38% for fill factor (FF)). Obviously, the increases in photovoltaic parameters of the PVK-based PSCs are mainly ascribed to enhanced *V_OC_* and FF.

**Fig. 2:**
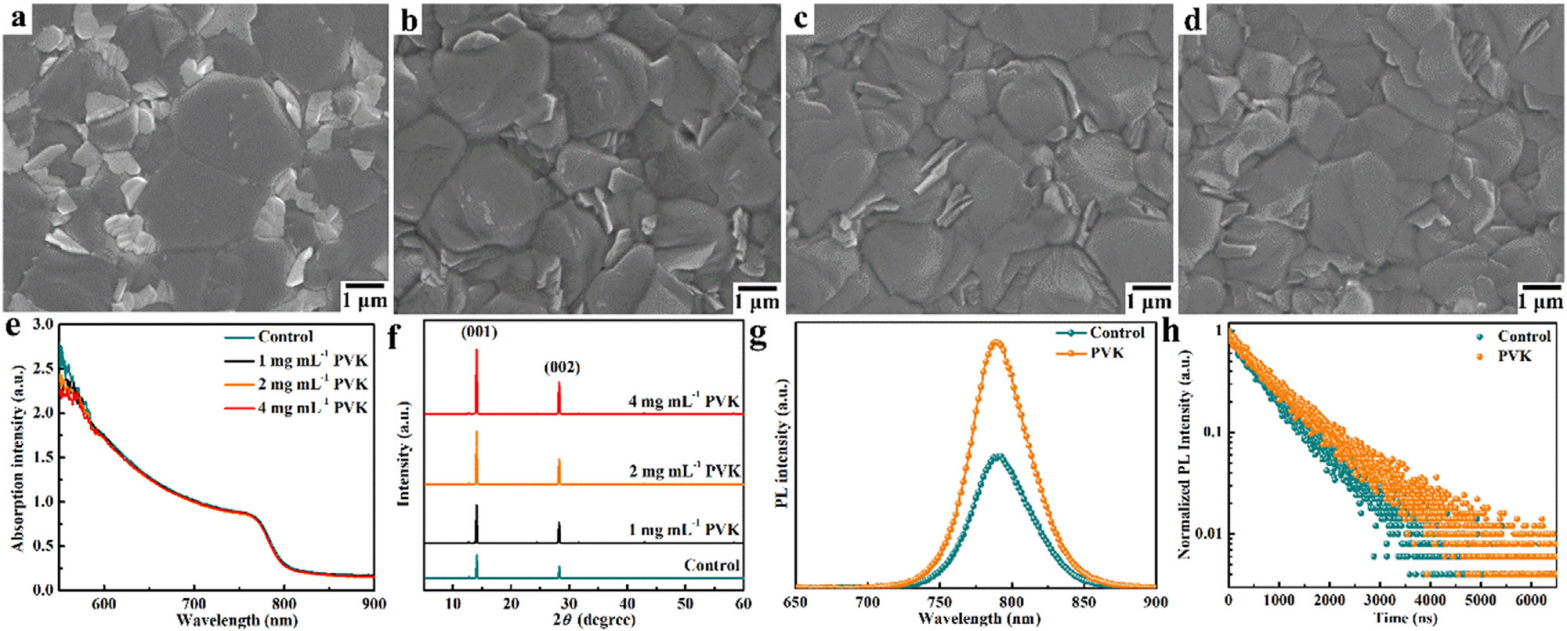
The SEM images of the control (a) and PVK-based perovskite films with the concentration of (b) 1 mg mL^−1^, (c) 2 mg mL^−1^, and (d) 4 mg mL^−1^. (e) UV–Vis absorption spectra and (f) XRD patterns of the perovskite films with different concentrations of PVK solution. (g) PL spectra and (h) TRPL spectra of the perovskite films without and with PVK layer.

To reveal the influence of PVK on surface morphology, the perovskite films are analyzed using SEM measurements. From Fig. 2a-d, the control perovskite film shows large grains and residual PbI_2_ with random distribution [21,36]. The surface of perovskite films can be covered with PVK layer after spin-coating PVK solution. The uniform PVK layer is formed by setting the PVK solution concentration to 2 mg mL^−1^, which is beneficial for passivating the surface of perovskite films. Meanwhile, further increasing PVK concentration, the thicker PVK layer can affect the hole transport [29]. Furthermore, the light-harvesting ability of ITO/SnO_2_/FA_0.95_Cs_0.05_PbI_3_ perovskite films is studied by UV–Vis absorption spectra, as shown in Fig. 2e. There are similar absorption spectra of perovskite films covered with different concentrations of PVK solution, indicating that PVK layer has no effect on absorption bandgap of perovskite films. Fig. 2f shows XRD patterns of ITO/SnO_2_/FA_0.95_Cs_0.05_PbI_3_ perovskite films. The main diffraction peaks at around 14.1° and 28.3° are ascribed to (001) and (002) planes of FA_0.95_Cs_0.05_PbI_3_ perovskite films, respectively [31]. Obviously, the crystallization intensity of the PVK-based perovskite films is improved, due to the passivation of PVK and the Ostwald ripening of perovskite grains [36]. Meanwhile, the residual PbI_2_ can be observed at around 12.7°. The peak intensity ratio of PbI_2_ to the (001) plane in the perovskite films decreased from 7.2% in the control perovskite film and 4.4% in the PVK-based perovskite film with 1 mg mL^−1^ to 2.6% in the PVK-based perovskite film with 2 mg mL^−1^. The PVK-based perovskite film with 4 mg mL^−1^ shows a similar ratio to the perovskite film with 2 mg mL^−1^. Therefore, the concentration of PVK solution should be set as 2 mg mL^−1^.

To confirm the interaction between functional group of PVK and perovskite, FTIR spectra of PVK, PbI_2_, and PVK-PbI_2_ are shown in Fig. S3. Vibration bands at around 677–778 cm^−1^ and 1427–1499 cm^−1^ are attributed to the bending vibration of −C−H and the stretching vibration of −C=C−, resulting from carbazole functional group of PVK [37,38]. The shift of characteristic peaks indicates the strong interaction between the carbazole functional group and uncoordinated Pb^2+^, owing to the more negative partial charge [25]. Then, interfacial charge transfer dynamics of ITO/FA_0.95_Cs_0.05_PbI_3_ perovskite films are elaborated by PL and TRPL spectra. Fig. 2g shows steady-state PL spectra of ITO/FA_0.95_Cs_0.05_PbI_3_ perovskite films. The PL intensity of the PVK-based perovskite film is larger than that of the control perovskite film, indicating reduced surface defects [39]. Thus, the surface of perovskite films is passivated by the PVK layer to improve the crystallization quality, which is consistent with XRD results. Furthermore, the TRPL of ITO/FA_0.95_Cs_0.05_PbI_3_ perovskite films are shown in Fig. 2h. TRPL spectra are fitted by a double exponential model to analyze the carrier transport and recombination characteristics [40,41]. The PVK-based perovskite film shows longer carrier lifetimes (τ_ave_ = 793.83 ns) than the control perovskite film (τ_ave_ = 640.15 ns). It is demonstrated that the non-radiative recombination is significantly restrained by the reduced surface defects, which is beneficial to increasing *V*_oc_ value.

**Fig. 3:**
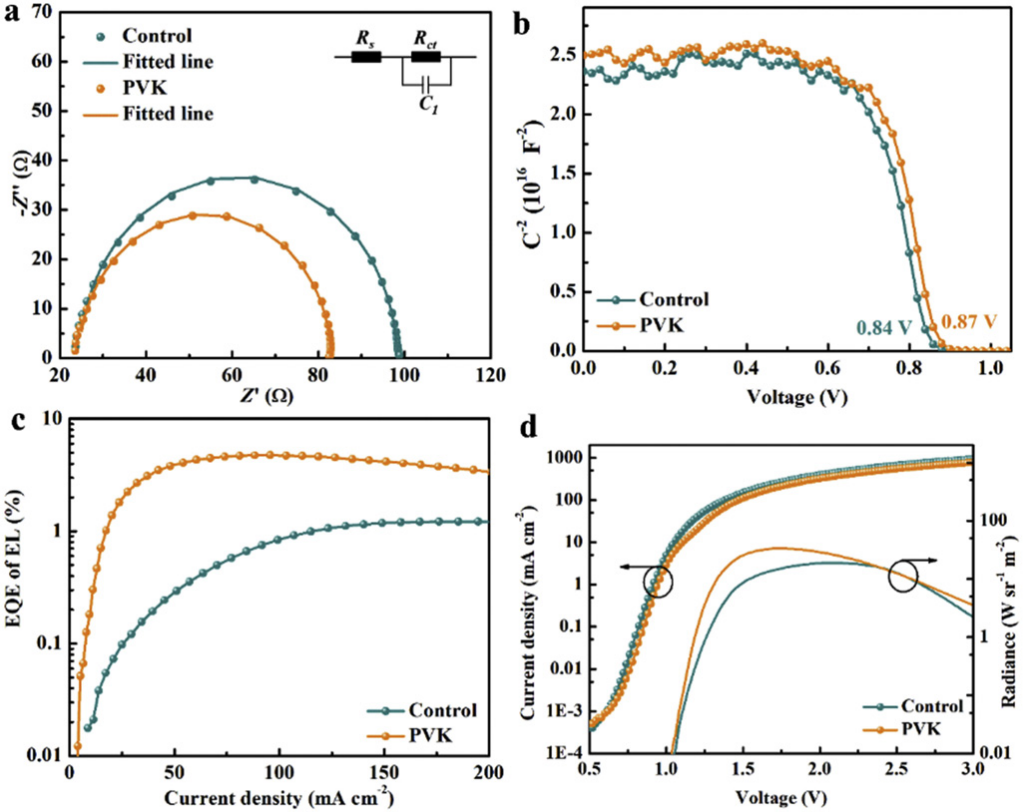
(a) Nyquist plots of PSCs measured in the dark (inset: the equivalent circuit). (b) Mott–Schottky plots of PSCs. (c) EQE of the electroluminescence as the function of current density. (d) J-V and radiance versus voltage curves of PSCs.

The trap density of the hole-only devices with the structure of ITO/PEDOT:PSS/perovskite/spiro-OMeTAD/MoO_3_/Ag is also analyzed using the SCLC method. Fig. S4 shows SCLC curves of the hole-only devices with and without the PVK layer. The trap-filled limit voltage (*V*_TFL_) value of the device with a PVK layer is 0.22 V, which is smaller than that of the device without a PVK layer (0.28 V). Generally, the trap density of the devices is proportional to the *V*_TFL_ value [42,43]. It can be seen that the PVK-based perovskite film displays the smaller trap density due to the passivation of PVK. The interface between perovskite film and spiro-OMeTAD layer is also investigated by cross-sectional SEM images and UPS spectra. From Fig. S5, the interfacial without pinholes is obtained by inducing the PVK layer. The UPS spectra of perovskite films are shown in Fig. S6. The valence band of the control perovskite film is −5.83 eV, which is lower than the HOMO energy level of the PVK-based perovskite film (−5.76 eV) [31]. It is demonstrated that the HOMO energy level of PVK is well aligned with the valance band of perovskite film, facilitating hole extraction and transport. The electrical properties of PSCs are studied by EIS, M-S curves, and EQE of electroluminescence. Fig. 3a shows Nyquist plots of PSCs under dark conditions with the applied bias voltage set to *V*_oc_. The semicircle in the high-frequency region represents the charge transfer resistance (*R_ct_*) in PSCs [44]. From the insert in Fig. 3a, the equivalent circuit with a contact resistance (*R_s_*) in series and a parallel phase element with constant resistance (*R_ct_* and *C*) is used to fit the Nyquist plots of PSCs. The *R_ct_* values of PSCs without and with PVK layer are 74.6 Ω and 59.5 Ω, respectively. The smaller *R_ct_* value for the PVK-based PSC is associated with the improved charge transfer. The hole transport and extraction process at the interface between perovskite film and spiro-OMeTAD layer is improved, which increases FF value [45,46]. The built-in potential (*V_bi_*) of PSCs is obtained by Mott–Schottky plots (Fig. 3b) [47,48]. The *V_bi_* value of the PVK-based PSC is 0.87 V, which is higher than that of the control PSC (0.84 V). The higher *V_bi_* value indicates the larger *V*_oc_ value, which is due to the increased driving force of the photogenerated carriers. The results are consistent with the results of the J-V curves.

According to the Shockley-Queisser limit, the EQE of the electroluminescence (*EQE_EL_*) in the dark under bias voltage can be applied to evaluate the recombination in PSCs [49,50]. Fig. S7 shows EL spectra of PSCs under different bias voltages operating as light-emitting diodes. The obvious emission peaks are observed at around 796 nm. The non-radiative open-circuit voltage (*V_OC_*) loss of PSCs is calculated by Eq. 1 [[51], [52], [53]].

(1) $$\Delta V_{OC}^{non-rad}=-\frac{k_{B}T}{e}\ln\left(EQE_{EL}\right)$$

where $\Delta V_{OC}^{non-rad}$ is the *V*_oc_ loss by the non-radiative recombination. *k_B_* is the Boltzmann constant. *T* is the Kelvin temperature. *e* is the elementary charge. The *EQE_EL_* value is determined by setting the injection current density to *J*_sc_. From Fig. 3c, the *EQE_EL_* value of the control PSC is 0.1% below the injection current density of 24.45 mA cm^−2^. The *EQE_EL_* value of the PVK-based PSC is 1.9% below the injection current density of 24.51 mA cm^−2^. The corresponding $\Delta V_{OC}^{non-rad}$ values of the control and PVK-based PSCs are 177 mV and 102 mV, respectively. This result demonstrates that the PVK-based PSC exhibits lower energy loss than the control PSC. The $\Delta V_{OC}^{non-rad}$ values are almost consistent with the J-V curves, while the *V*_oc_ values of PSCs without and with the PVK layer are 1.11 V and 1.17 V, respectively. Fig. 3d shows the J-V and radiance-voltage curves of PSCs. The PVK-based PSC also has a larger radiance of 33.42 W sr^−1^ m^−2^ than the control PSC (18.95 W sr^−1^ m^−2^). The results indicate that the non-radiative recombination process is also suppressed by introducing the PVK layer.

**Fig. 4:**
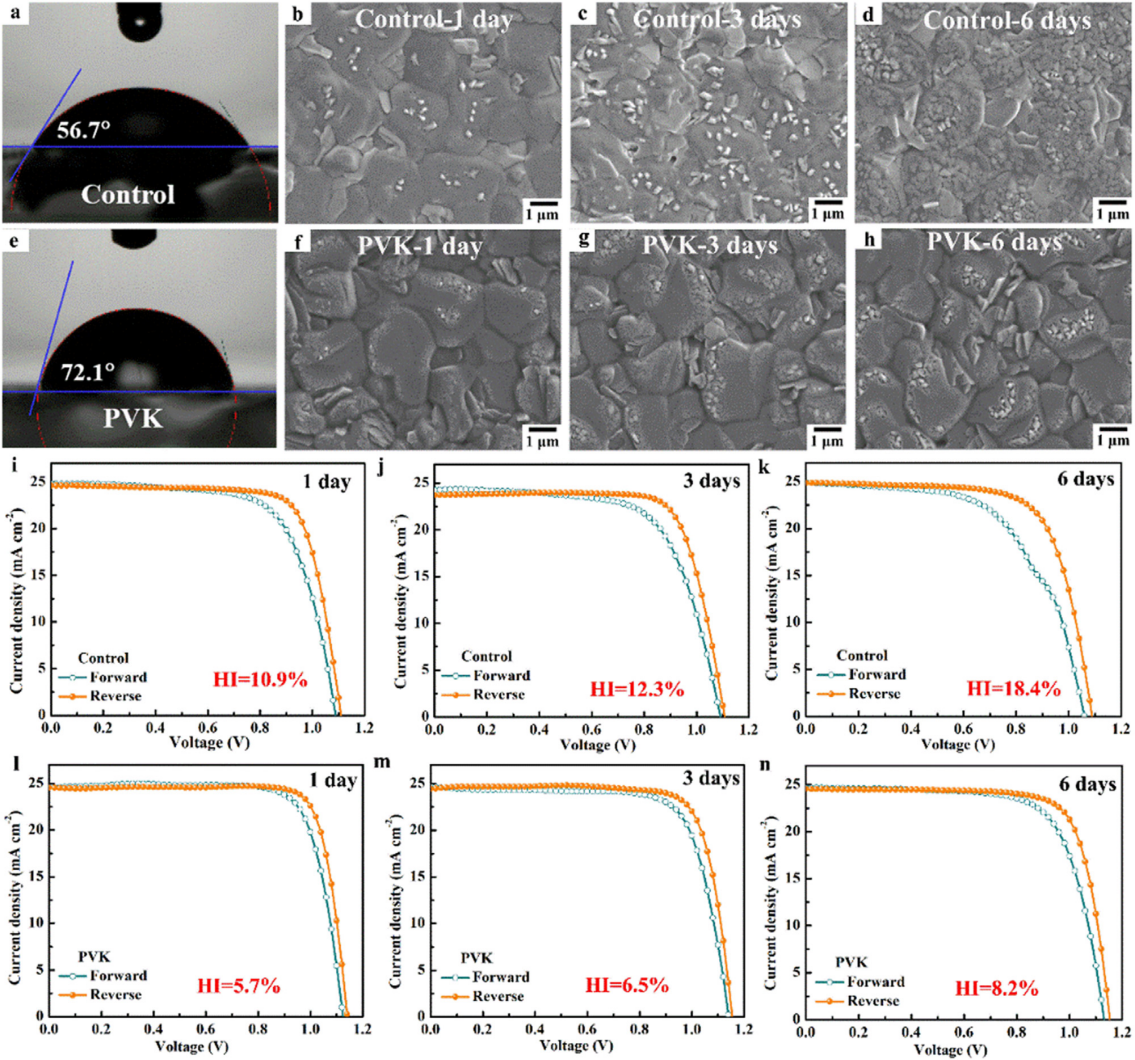
Static contact angles of (a) the control perovskite film and (e) the PVK-based perovskite film. SEM images of the control perovskite films and PVK-based perovskite films with different degradation times under ∼50% RH: (b) and (f) 1 day, (c) and (g) 3 days, and (d) and (h) 6 days. The J*-*V curves of PSCs without and with PVK layer: (i) and (l) 1 day, (j) and (m) 3 days, (k) and (n) 6 days.

The hydrophobic properties of the perovskite films play a vital role in avoiding water degradation and improving the reproducibility of PSCs prepared in ambient air. Thus, we conduct water contact angle measurements on the perovskite films and characterize their microscopic morphology and device performance of the PSCs. As shown in Fig. 4a, e and Fig. S8, the PVK-based perovskite film has a larger water contact angle than the control perovskite film. The contact angle gradually increases with increasing PVK concentration, due to the uniform PVK layer with hydrophobic properties [29]. The optimized PVK layer has a contact angle of 72.1° Therefore, the PVK-based perovskite film can improve the moisture resistance. To demonstrate this, we investigate the humidity effect on the microstructure of the perovskite films. The fabricated FA_0.95_Cs_0.05_PbI_3_ perovskite films are degraded under 50% RH condition for 1 day, 3 days and 6 days and then used to prepare PSCs. As shown in Fig. 4b-d, more and more PbI_2_ is formed in the control perovskite film with increasing degradation time. When degradation time is up to 6 days, the surface of the control perovskite films is almost degraded. On the contrary, the surface of the PVK-based perovskite film does not change significantly with the increase of degradation time (Fig. 4f-h). Furthermore, the perovskite films with different degradation times are used to fabricate PSCs. The photovoltaic performance of the corresponding PSCs also demonstrates a similar outcome. As shown in Fig. 4i-n and Table S3, the PCE of the control PSC decreases from 20.69% for 1 day and 19.93% for 3 days to 19.05% for 6 days. With the increase of degradation time, the HI of the control PSC increased from 10.9% for 1 day to 18.4% for 6 days. Meanwhile, the PCE of the PVK-based PSC slightly decreases from 22.87% for 1 day and 22.30% for 3 days to 21.64% for 6 days. The HI of the PVK-based PSC increases from 5.7% for 1 day to 8.2% for 6 days. Therefore, due to their hydrophobic property, the FA_0.95_Cs_0.05_PbI_3_ perovskite films are protected under the moisture ambient air by introducing a PVK layer. Thereby, it is proved that the hydrophobicity of PVK is extremely beneficial to improving the photovoltaic performance and reproducibility of PSCs.

**Fig. 5:**
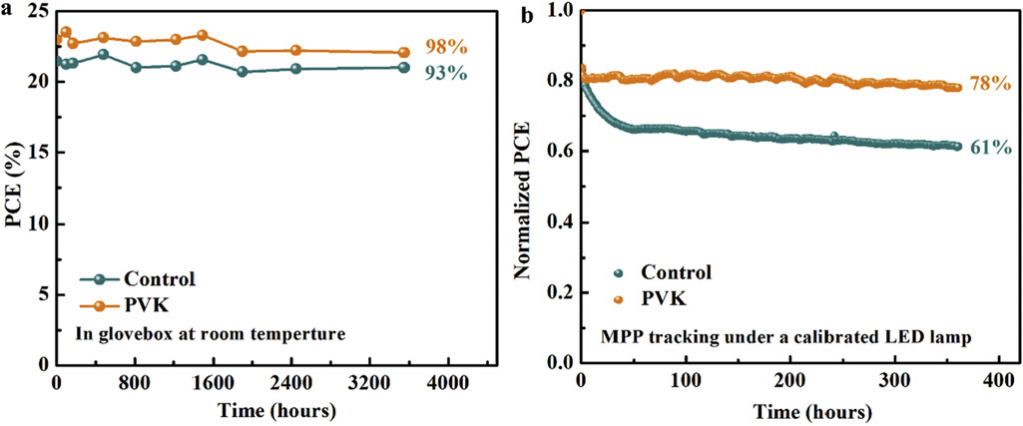
(a) The long-term storage stability in an argon-filled glovebox. (b) The operational stability of PSCs under MPP conditions in an argon-filled glovebox.

In addition, the environmental stability of unencapsulated PSCs is also studied. Fig. 5a shows that the PVK-based PSC in an argon-filled glovebox remains 98% of the initial PCE even after about 3550 h at room temperature. Fig. 5b shows the operational stability of PSCs under MPP conditions in an argon-filled glovebox. The control PSC has a rapid loss of PCE, while the PVK-based PSC still has 78% of the initial PCE after approximately 360 h at room temperature. Therefore, the PVK-based PSC exhibits excellent environmental stability.

## 4 Conclusion

In summary, we have successfully prepared PSCs in an ambient air environment with excellent photovoltaic performance and stability by introducing a PVK hydrophobic layer. The uniform PVK layer passivates the defects and facilitates hole extraction and transport, leading to a small non-radiative open-circuit voltage loss. As a result, the champion PVK-based PSC achieved a high PCE of 23.27%, higher than that of the control PSC (20.94%). Furthermore, the large water contact angle of the PVK-based perovskite film indicates the enhanced capability of moisture tolerance under RH ∼ 50% ambient conditions. The PVK-based PSC can maintain 21.64% PCE with a small hysteresis index (HI) after the corresponding perovskite film is degraded for 6 days. Moreover, the PVK-based PSC exhibits remarkable stability, remaining 98% of the initial PCE after about 3550 h in an argon-filled glovebox. This work offers a novel method for producing high-efficiency PSCs in a humid environment.

## Declaration of competing interest

The authors declare that they have no conflicts of interest in this work.

## Appendix Supplementary materials

Image, application 1
