# Supplementary material for: Hydrophobic interface layer improves the moisture tolerance and efficiency of ambient air-processed perovskite solar cells
Source: Fundam Res. 2024 Dec 27;6(2):1071–7. doi: 10.1016/j.fmre.2024.12.013 (PMC13069614; doi:10.1016/j.fmre.2024.12.013)
Supplement: Supplementary file 1 [file mmc1.docx]

**Supporting Information**

# Hydrophobic interface layer improves the moisture tolerance and efficiency of ambient air-processed perovskite solar cells

Wanjie Yin ^a^, Huiming Luo ^b^, Ligang Yuan ^c^, Yuxuan Sun ^a^, Xiao Yang ^a,^*, Longyan Zhang ^a^, Yong Peng ^d^, Qing-Song Jiang ^a,^*

*^a^ Faculty of Electronic Information Engineering, Huaiyin Institute of Technology, Huai’an 223003, China*

*^b^ Institute for Materials Discovery, University College London, Malet Place, London, WC1E 7JE, UK*

*^c^ Key Laboratory for Optoelectronic Information Perception and Instrumentation of Jiangxi Province, Key Laboratory of Nondestructive Testing Ministry of Education, School of the Testing and Photoelectric Engineering, Nanchang Hangkong University, Nanchang, 330063 China*

*^d^ State Key Lab of Advanced Technology for Materials Synthesis and Processing, Wuhan University of Technology, Wuhan 430070, China*

** Corresponding author: yangxiao@hyit.edu.com (X. Yang), jiangqingsong05@hyit.edu.cn (Q.-S. Jiang)*


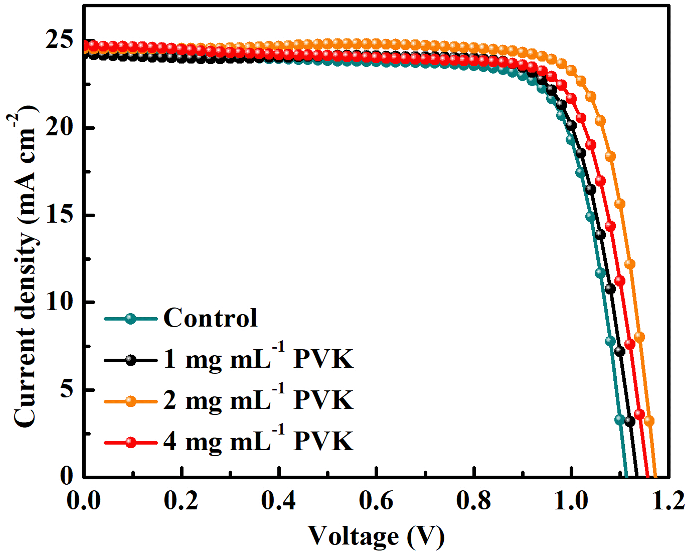


**Fig. S1.** *J-V* curves of PSCs based on PVK layer with different concentrations.


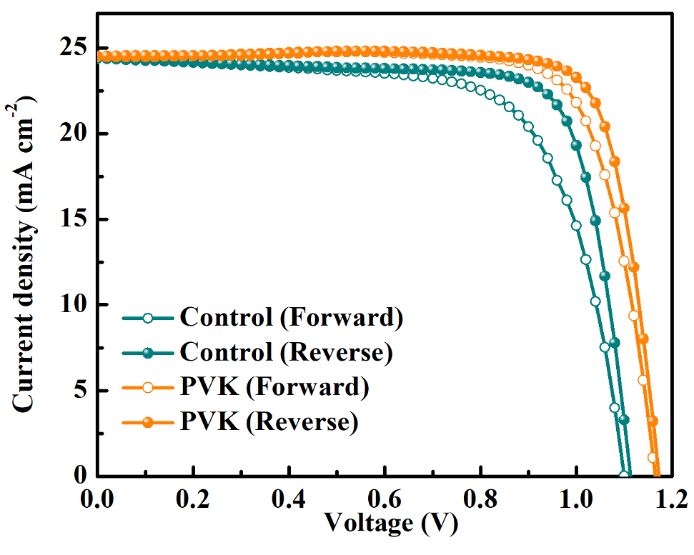


**Fig. S2.** *J*-*V* curves of PSCs without and with PVK layer in forward and reverse scan measurements.





**Fig. S3.** FTIR spectra of PVK, PbI_2_, and PVK-PbI_2_.





**Fig. S4**. SCLC curves of the hole-only devices without and with PVK layer.





**Fig. S5.** Cross-sectional SEM images of PSCs (a) without and (b) with PVK layer.





**Fig. S6.** UPS spectra of the perovskite film without and with PVK layer.


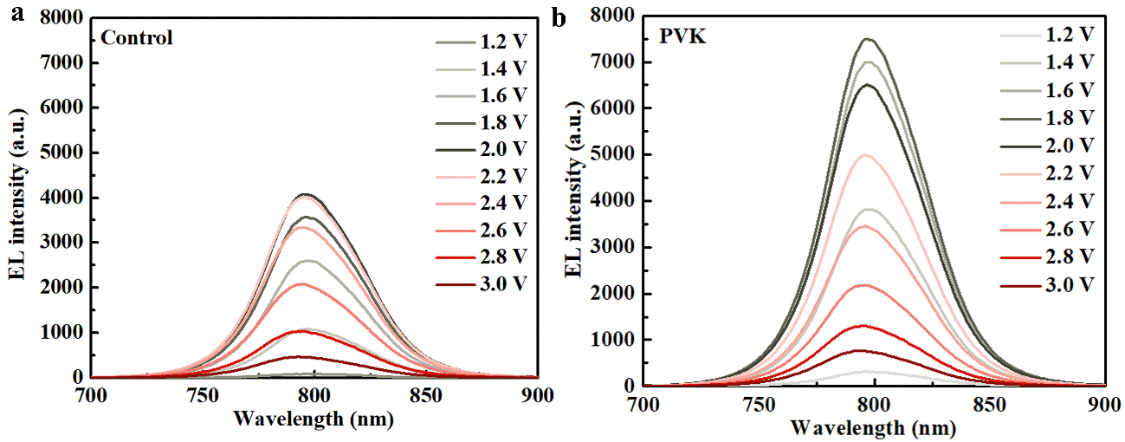


**Fig. S7.** EL spectra of the PSCs (a) without and (b) with PVK layer under different bias voltages operating as LEDs.





Fig. S8. Static contact angles of the perovskite films with (a) 1 mg mL^−1^ PVK and (b) 4 mg mL^−1^ PVK.

**Table S1**. Photovoltaic parameter of PSCs without and with PVK layer in forward and reverse scan measurements.

| PSCs | V_oc_ (V) | J_sc_ (mA cm^−2^) | FF (%) | PCE (%) | HI (%) |
| --- | --- | --- | --- | --- | --- |
| Control-forward | 1.10 | 24.37 | 69.12 | 18.53 | 11.5 |
| Control-reverse | 1.11 | 24.45 | 77.16 | 20.94 |  |
| PVK-forward | 1.16 | 24.48 | 78.14 | 22.19 | 4.6 |
| PVK-reverse | 1.17 | 24.51 | 81.15 | 23.27 |  |

**Table S2.** The average photovoltaic parameter of the champion PSCs without and with PVK layer.

| PSCs | *V*_oc_ (V) | *J*_sc_ (mA cm^−2^) | FF (%) | PCE (%) |
| --- | --- | --- | --- | --- |
| Control | 1.09 ± 0.02 | 24.31 ± 0.26 | 75.89 ± 2.09 | 20.12 ± 0.59 |
| PVK | 1.15 ± 0.01 | 24.88 ± 0.45 | 77.69 ± 2.38 | 22.21 ± 0.71 |

**Table S3.** Photovoltaic parameter of PSCs fabricated by the perovskite films with different degradation times in forward and reverse scan measurements.

| Day | PSCs | *V*_oc_ (V) | *J*_sc_ (mA cm^−2^) | FF (%) | PCE (%) | HI (%) |
| --- | --- | --- | --- | --- | --- | --- |
| 1 | Control-forward | 1.09 | 24.77 | 68.26 | 18.43 | 10.9 |
|  | Control-reverse | 1.11 | 24.59 | 75.80 | 20.69 |  |
| 3 | Control-forward | 1.09 | 24.24 | 66.16 | 17.48 | 12.3 |
|  | Control-reverse | 1.11 | 23.70 | 75.76 | 19.93 |  |
| 6 | Control-forward | 1.06 | 24.91 | 58.85 | 15.54 | 18.4 |
|  | Control-reverse | 1.09 | 24.87 | 70.27 | 19.05 |  |
| 1 | PVK-forward | 1.12 | 24.58 | 78.35 | 21.57 | 5.7 |
|  | PVK-reverse | 1.14 | 24.55 | 81.72 | 22.87 |  |
| 3 | PVK-forward | 1.14 | 24.65 | 74.23 | 20.86 | 6.5 |
|  | PVK-reverse | 1.15 | 24.45 | 79.31 | 22.30 |  |
| 6 | PVK-forward | 1.13 | 24.64 | 71.32 | 19.86 | 8.2 |
|  | PVK-reverse | 1.15 | 24.55 | 76.65 | 21.64 |  |
